# Distribution and Community Composition of Anammox Bacteria in Shallow Groundwater of the Kathmandu Valley, Nepal

**DOI:** 10.1264/jsme2.ME20143

**Published:** 2021-03-09

**Authors:** Mai Nakano, Tatsuru Kamei, Bijay Man Shakya, Takashi Nakamura, Yasuhiro Tanaka, Eiji Haramoto, Tadashi Toyama, Futaba Kazama

**Affiliations:** 1 Integrated Graduate School of Medicine, Engineering and Agricultural Science, University of Yamanashi, 4–3–11 Takeda, Kofu, Yamanashi 400–8511, Japan; 2 Interdisciplinary Center for River Basin Environment, University of Yamanashi, 4–3–11 Takeda, Kofu, Yamanashi 400–8511, Japan; 3 Department of Environmental Sciences, Faculty of Life and Environment Sciences, University of Yamanashi, 4–4–37 Takeda, Kofu, Yamanashi 400–8510, Japan

**Keywords:** anammox bacteria, distribution, groundwater, Kathmandu Valley

## Abstract

The abundance and diversity of anaerobic ammonium oxidation (anammox) bacteria were assessed in 152 groundwater samples in the Kathmandu Valley, Nepal. Anammox bacterial 16S rRNA genes were detected in 54% (37/68) of samples collected in the dry season at 1.6×10^5^–8.8×10^6^ copies L^–1^, and in 60% (50/84) of samples collected in the wet season at 4.3×10^4^–1.2×10^7^ copies L^–1^. The 16S rRNA genes of “*Candidatus* Brocadia”, “*Candidatus* Anammoxoglobus”, and five new deduced anammox bacterial phylotypes were detected in the shallow groundwater samples. Diverse anammox bacteria were broadly distributed in the shallow groundwater aquifer of the Kathmandu Valley.

Anaerobic ammonium oxidation (anammox) is a key microbe-driven nitrogen (N) cycle process, in which ammonium (NH_4_^+^) is oxidized with nitrite (NO_2_^–^) to produce nitrogen gas (N_2_) under anoxic conditions (Jetten, 2008). Anammox has been observed in various natural environments, including marine (Babbin *et al.*, 2014), estuarine (Hou *et al.*, 2013), freshwater (Yang *et al.*, 2017), soil (Shen *et al.*, 2013), and freshwater sediment (Osaka *et al.*, 2012).

Groundwater is an important resource for domestic and drinking water; however, the contamination of groundwater by NH_4_^+^-N is a serious issue in some parts of the world (Nguyen *et al.*, 2009; Groeschke *et al.*, 2017). Anammox bacteria play an important role in the N cycle and N removal in freshwater aquifers (Moore *et al.*, 2011). Nevertheless, limited information is currently available on the distribution and diversity of anammox bacteria in groundwater and, to the best of our knowledge, their distribution and diversity in the groundwater basin has not yet been examined.

We selected the Kathmandu Valley (area 664‍ ‍km^2^; approximately 25‍ ‍km long from north to south and 30‍ ‍km wide) in Nepal as a case study location because its groundwaters are widely contaminated by NH_4_^+^-N (Shakya *et al.*, 2019). The objective of the present study was to assess the distribution and diversity of anammox bacteria in order to obtain a more detailed understanding of the contribution of anammox to N removal in the groundwater basin. The shallow groundwater samples and groundwater-extracted DNA samples used in the present study were the same as those previously prepared and reported by Malla *et al.* (2018). In the dry season (February and March), groundwater samples (*n*=68) were collected from shallow dug wells in the gravel subsurface (*n*=24) and clay subsurface (*n*=44). In the wet season (August and September), shallow groundwater samples (*n*=84) were collected in the gravel subsurface (*n*=29) and clay subsurface (*n*=55) (Fig. S1, Table S1 and S2). To quantify the 16S rRNA genes of anammox bacteria and total bacteria, qPCR was performed using the primer sets AMX-368F (Schmid *et al.*, 2003) and Amx-667R (van der Star *et al.*, 2007) for the former and the primer set Eub-515F and Eub-806R (Caporaso *et al.*, 2011) for the latter. The thermal conditions of PCR are shown in Table S3. DNA samples extracted from groundwater at Khumaltar (Table S4), a clay subsurface area, and Nayachok (Budhanilkantha) (Table S4), a gravel subsurface area, were used in the phylogenetic analysis. Anammox bacterial 16S rRNA genes were amplified using the Pla46F (Neef *et al.*, 1998) and Amx820R primers (Schmid *et al.*, 2000). The Pla46F and Amx 820R primers were previously reported to be an effective primer set that widely detected anammox bacteria (Nakajima *et al.*, 2008). Amplified DNA fragments were cloned into *Escherichia coli* strain DH5α using a pMD19 T-vector system (Takara Bio). A PCR-Restriction Fragment Length Polymorphism (RFLP) analysis and clone library analysis were performed as previously reported (Tanaka *et al.*, 2012). The phylogenetic tree was constructed using Clustal X (http://www.clustal.org/clustal2/). The DDBJ/EMBL/GenBank accession numbers of the 16S rRNA gene sequences of clones are LC553643 to LC553674.

As shown in Table 1, in the dry season, the 16S rRNA genes of anammox bacteria were detected in 58% (14/24) of shallow groundwater samples in the gravel subsurface and in 52% (23/44) of those in the clay subsurface (Table 1). Anammox bacterial 16S rRNA gene abundance ranged between 1.7×10^5^ and 7.6×10^6^ copies L^–1^ in groundwater in the gravel subsurface and between 1.6×10^5^ and 8.8×10^6^ copies L^–1^ in those in the clay subsurface. In the wet season, the 16S rRNA genes of anammox bacteria were detected in 69% (20/29) of shallow groundwater samples in the gravel subsurface and in 55% (30/55) of those in the clay subsurface, with 1.2×10^5^ to 1.2×10^7^ copies L^–1^ and between 4.3×10^4^ and 3.1×10^6^ copies L^–1^ in groundwater samples in gravel and clay subsurfaces, respectively (Table 1). Anammox bacterial 16S rRNA gene abundance (copies L^–1^) was as high as that in other freshwater samples (Sun *et al.*, 2014; Zhang *et al.*, 2017). These results suggest that anammox bacteria were widely distributed throughout the Kathmandu Valley groundwater basin in the dry and wet seasons (Fig. 1).

Water temperature, pH, and NH_4_^+^-N concentrations have been suggested to affect the abundance of anammox bacteria in freshwater environments (Sun *et al.*, 2013; Sun *et al.*, 2014; Yang *et al.*, 2017). However, in the present study, anammox bacteria were widely detected in the shallow groundwater samples with a temperature range of 15.5–32.0°C, pH range of 5.7–7.3, and NH_4_^+^-N concentration range of 0–5.6‍ ‍mg L^–1^, and their abundance did not correlate with any of these water properties (R statistical software version 3.6.0, *P*<0.05, Table S5). The dissolved oxygen (DO) concentration was an important environmental parameter. However, the relationship between the anammox bacterial 16S rRNA gene and DO was not investigated because the DO value was not assessed (Shakya *et al.*, 2019). Although the underlying reasons were not elucidated in the present study, the shallow groundwater basin in the Kathmandu Valley appears to be a consistent habitat for anammox bacteria.

Based on the results of the PCR-RFLP and clone library analyses for the anammox bacterial 16S rRNA gene, 143 clones were divided into 32 RFLP groups (Table S6). A phylogenetic analysis identified eight groups: Brocadia, Anammoxoglobus, Clusters 1, 2, 3, 4, 5, and 6 (Fig. S2). The Brocadia group comprised “*Candidatus* Brocadia” and two RFLP groups (three clones), while the Anammoxoglobus group consisted of “*Candidatus* Anammoxoglobus” and RFLP18 (five clones). Cluster 1 comprised 16 RFLP groups including 66 clones. Clusters 2 and 3 each consisted of a single RFLP group, RFLP23 (46 clones) and RFLP24 (1 clone), respectively. Cluster 4 comprised three RFLP groups (13 clones), Cluster 5 consisted of six RFLP groups (seven clones), and Cluster 6 had two RFLP groups (two clones). “*Ca.* Brocadia” is recognized as a major anammox group in non-saline environments, such as freshwater sediments, freshwater, soils, and groundwater, while “*Ca.* Anammoxoglobus” has also been frequently detected in non-saline environments, including soil, freshwater, estuaries, and groundwater (Moore *et al.*, 2011; Sonthiphand *et al.*, 2014). In the present study, “*Ca.* Brocadia” and “*Ca.* Anammoxoglobus” were also present, but not dominant, in the shallow groundwater samples at Nayachok and Khumaltar. Clusters 1 and 2 were the dominant groups in groundwater at both Nayachok and Khumaltar in the dry and wet seasons (Fig. S3). These results suggest that diverse anammox bacteria are present in the shallow groundwater of the Kathmandu Valley. Anammox bacteria in Clusters 1 and 2 may exhibit better adaptability in the groundwater. Further studies are needed to elucidate the relationships between the anammox bacterial community and environmental factors (*e.g.*, NH_4_^+^-N, NO_2_^–^-N, temperature, pH, DO, minerals, metals, and organic carbons) in groundwater in more detail.

In conclusion, various anammox bacteria were widely distributed throughout the shallow groundwater basin in the Kathmandu Valley during the wet and dry seasons. Therefore, anammox bacteria may contribute to N removal in this groundwater basin.

## Citation

Nakano, M., Kamei, T., Man Shakya, B., Nakamura, T., Tanaka, Y., Haramoto, E., et al. (2021) Distribution and Community Composition of Anammox Bacteria in Shallow Groundwater of the Kathmandu Valley, Nepal. *Microbes Environ ***36**: ME20143.

https://doi.org/10.1264/jsme2.ME20143

## Supplementary Material

Supplementary Material

## Figures and Tables

**Fig. 1.**
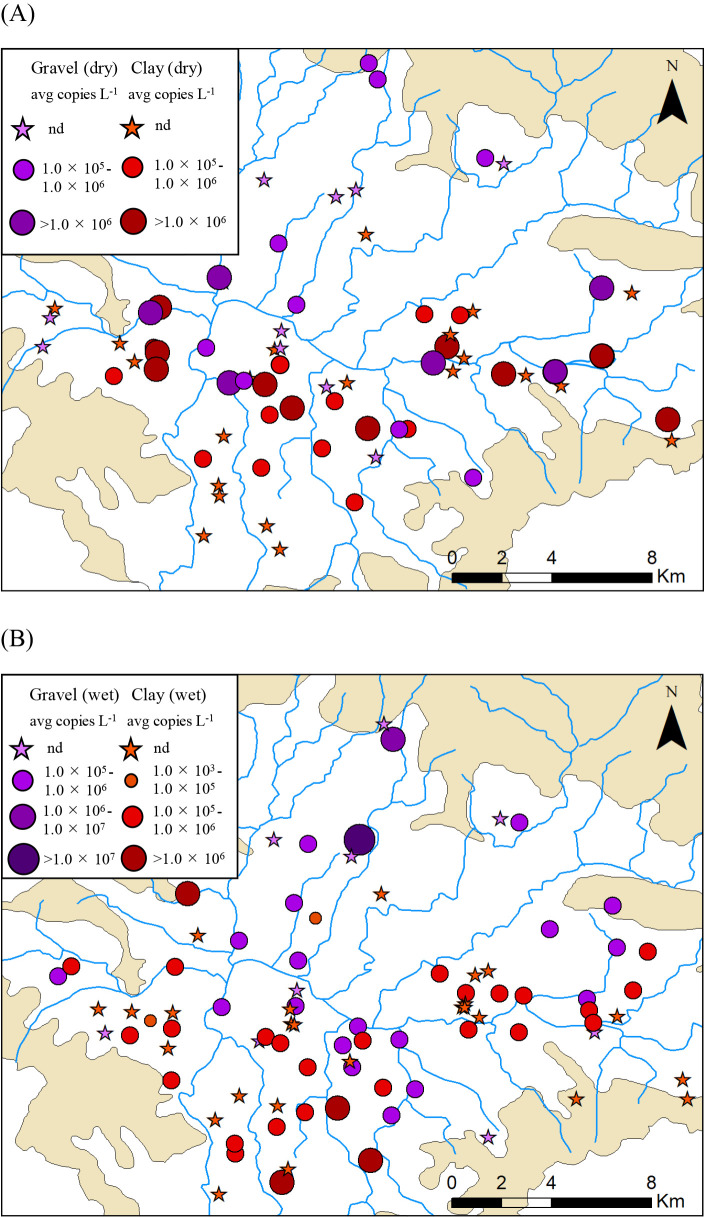
Spatial distribution and abundance of anammox bacteria in the groundwater of shallow dug wells in the Kathmandu Valley during dry (A) and wet (B) seasons.

**Table 1.** Detection and abundance of 16S rRNA genes of anammox bacteria and total bacteria in groundwater samples from shallow dug wells

Season	Sample type	Samples with the gene relative to all samples (detection %)	Anammox bacterial 16S rRNA gene abundance (copies L^–1^)				Total bacterial 16S rRNA gene abundance (copies L^–1^)		
			Min	Max	Ave*^a^*		Min	Max	Ave*^a^*
Dry season	Gravel subsurface	14/24 (58%)	1.7×10^5^	7.6×10^6^	1.4×10^6^±1.9×10^6^		6.3×10^6^	1.7×10^10^	1.9×10^9^±3.9×10^10^
	Clay subsurface	23/44 (52%)	1.6×10^5^	8.8×10^6^	1.5×10^6^±1.8×10^6^		1.2×10^7^	7.3×10^10^	3.3×10^9^±1.1×10^10^
Wet season	Gravel subsurface	20/29 (69%)	1.2×10^5^	1.2×10^7^	9.4×10^5^±2.5×10^6^		3.6×10^6^	1.0×10^11^	5.0×10^9^±1.9×10^10^
	Clay subsurface	30/55 (55%)	4.3×10^4^	3.1×10^6^	5.3×10^5^±7.3×10^5^		3.3×10^6^	3.0×10^10^	3.6×10^9^±4.9×10^9 ^*^b^*
	Total	87/152 (57%)	4.3×10^4^	1.2×10^7^			3.3×10^6^	1.0×10^11^	

*^a^* Values are means±SD. *^b^* Significantly higher (Student’s *t*-test, *P*<0.05) than total bacterial 16S rRNA gene abundance in dry season (gravel subsurface and clay subsurface) samples and wet season (gravel subsurface) samples.
