# Supplementary material for: Distribution and Community Composition of Anammox Bacteria in Shallow Groundwater of the Kathmandu Valley, Nepal
Source: Microbes Environ. 2021 Mar 9;36(1):ME20143. doi: 10.1264/jsme2.ME20143 (PMC7966944; doi:10.1264/jsme2.ME20143)
Supplement: Supplementary file 1 — Supplementary Material [file 36_20143_s1.pdf]

Nakano *et al*. Figure S1

(A)

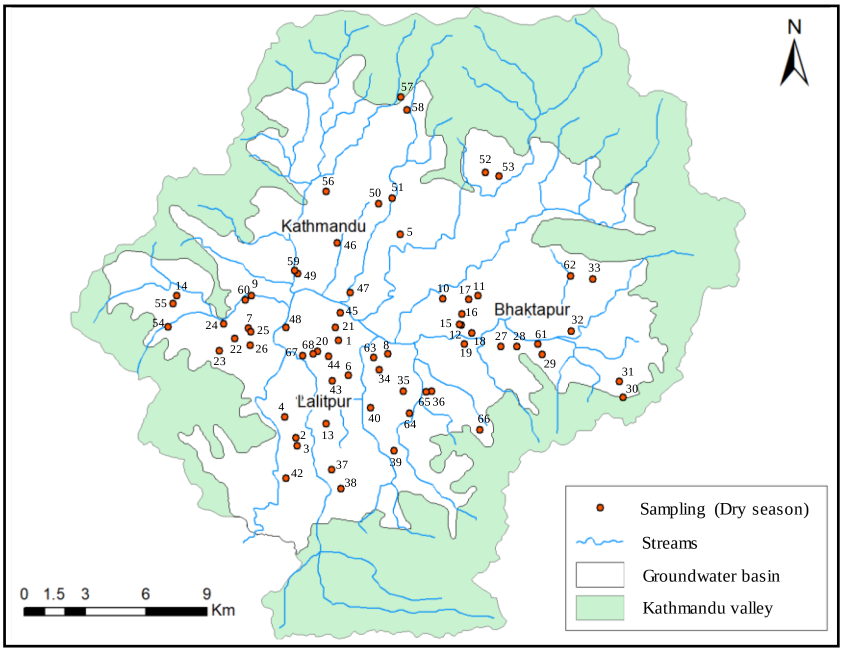

(B)

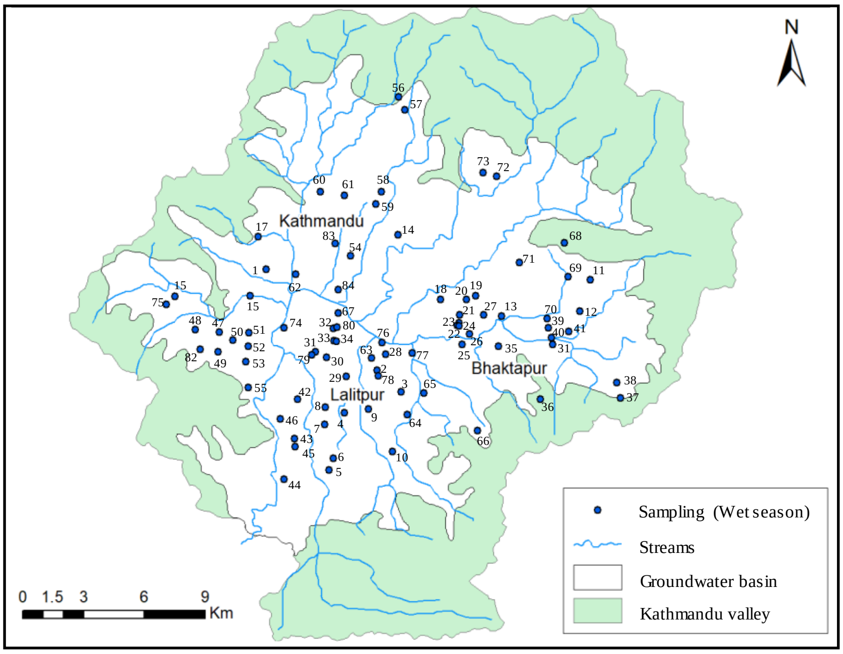

Fig. S1. Groundwater sampling locations in shallow dug wells in the Kathmandu Valley: (A) dry season, (B) wet season.

Nakano *et al.* Figure S2

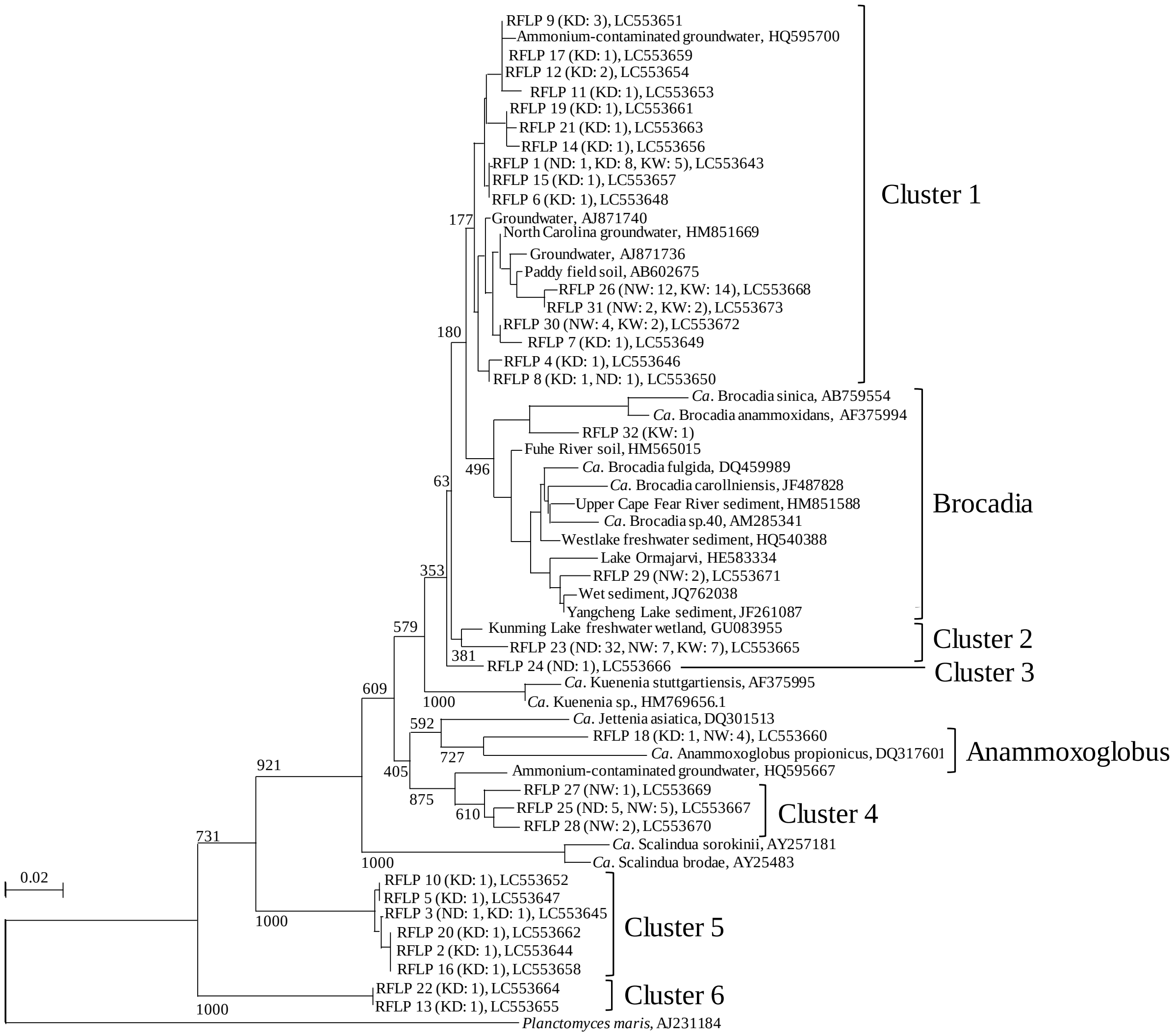

Fig. S2. Phylogenetic tree showing relationships of anammox bacteria-related 16S rRNA gene fragments in the clone libraries of groundwater samples from Khumaltar and Nayachok with known anammox bacterial DNA sequences in the GenBank database. The tree was constructed with Clustal X using the neighbor-joining method. Bootstrap node support values higher than 50% are shown (*n*=1,000 replicates). Representative clones of each RFLP group obtained in this study are indicated. In parentheses following the RFLP number are the sample name (ND, Nayachok in dry season; NW, Nayachok in wet season; KD, Khumaltar in dry season; KW, Khumaltar in wet season) and the number of clones of that RFLP group.

Nakano *et al*. Figure S3

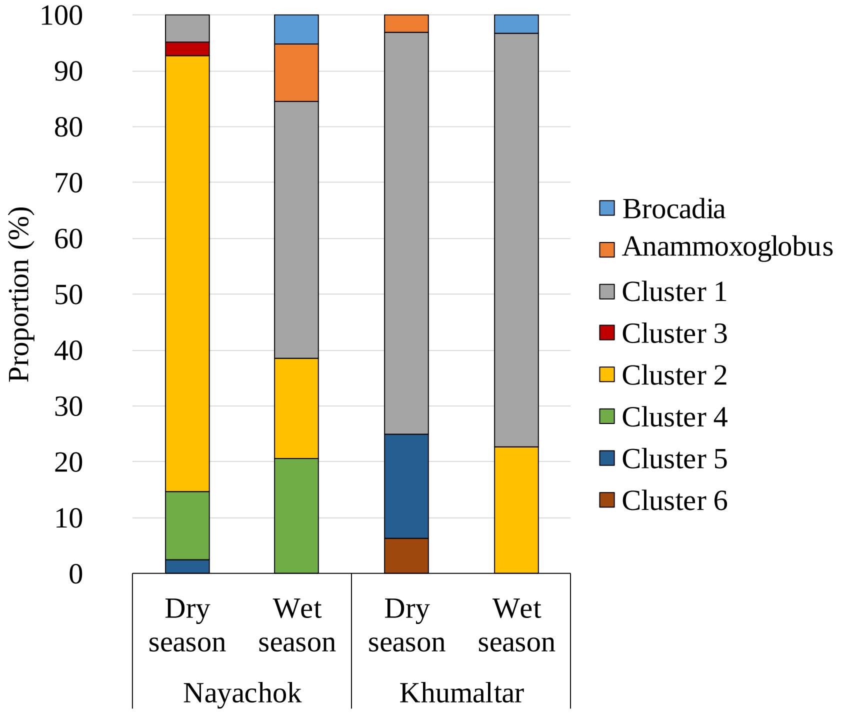

Fig. S3. Community compositions of anammox bacteria in shallow groundwater samples from Nayachok and Khumaltar in the dry and wet seasons.

Table S1. Physicochemical properties of groundwater samples collected from shallow dug wells in the dry season

No.	Sample ID	Sample type (subsurface)	Anammox bacterial 16S rRNA gene abundance (copies L^{-1})[a]	Total bacterial 16S rRNA gene abundance (copies L^{-1})[a]	NH_4^+-N (mg L^{-1})[b]	NO_3^--N (mg L^{-1})[b]	Water Temperature (°C)[b*]	pH[b*]
1	KTM 118	Clay	2.0×10^5	3.7×10^8	0.9	16.7	18.0	6.9
2	KTM 121	Clay	nd	3.5×10^7	0.3	0.6	18.0	9.5
3	KTM 122	Clay	nd	7.1×10^7	nd	1.2	17.6	7.1
4	KTM 124	Clay	7.7×10^5	2.5×10^8	2.8	3.9	16.5	7.0
5	KTM 125	Gravel	nd	6.9×10^8	nd	nd	16.2	7.1
6	KTM 133	Gravel	4.1×10^5	3.4×10^9	nd	11.4	20.3	6.2
7	KTM 135	Gravel	5.1×10^5	7.8×10^8	nd	nd	17.5	6.9
8	KTM 137	Clay	nd	2.5×10^8	nd	5.6	25.0	6.9
9	KTM 140	Clay	2.3×10^6	1.4×10^8	nd	25.6	19.0	6.8
10	KTM 144	Clay	2.0×10^5	2.2×10^8	0.2	17.6	17.5	6.5
11	KTM 147	Gravel	1.8×10^5	2.1×10^9	0.2	1.0	19.1	6.8
12	KTM 149	Gravel	nd	1.9×10^8	3.3	nd	16.8	7.5
13	KTM 156	Clay	nd	5.5×10^9	nd	2.5	17.2	7.0
14	KTM 158	Clay	2.8×10^6	1.3×10^8	nd	nd	18.4	6.8
15	KTM 163	Clay	6.5×10^5	1.5×10^8	nd	4.4	17.1	5.9
16	KTM 164	Clay	nd	1.2×10^8	nd	1.9	16.7	7.3
17	KTM 166	Clay	1.6×10^5	2.4×10^7	0.2	26.3	17.4	6.5
18	KTM 167	Clay	2.3×10^5	1.8×10^8	1.6	2.5	17.7	6.6

Nakano *et al*. Table S1

No.	Sample ID	Sample type (subsurface)	Anammox bacterial 16S rRNA gene abundance (copies L^{-1})[a]	Total bacterial 16S rRNA gene abundance (copies L^{-1})[a]	NH_4^+-N (mg L^{-1})[b]	NO_3^--N (mg L^{-1})[b]	Water Temperature (°C)[b*]	pH[b*]
19	KTM 171	Gravel	nd	1.0×10^{9}	nd	3.8	19.3	6.4
20	KTM 172	Gravel	nd	3.5×10^{8}	nd	1.7	17.5	5.8
21	KTM 174	Gravel	1.7×10^{5}	5.4×10^{7}	nd	1.2	18.0	6.1
22	KTM 175	Gravel	nd	3.0×10^{7}	0.2	3.2	16.4	6.0
23	KTM 182	Gravel	nd	1.0×10^{7}	nd	0.5	17.9	7.6
24	KTM 183	Gravel	nd	5.0×10^{8}	3.9	0.5	15.9	6.5
25	KTM 184	Clay	nd	1.6×10^{8}	nd	0.2	17.4	7.0
26	KTM 191	Gravel	nd	6.3×10^{6}	2.2	3.8	18.3	7.0
27	KTM 193	Gravel	5.4×10^{5}	3.1×10^{9}	nd	1.9	17.8	6.9
28	KTM 194	Gravel	6.0×10^{5}	1.8×10^{9}	1.8	13.4	17.8	6.4
29	KTM 201	Clay	3.3×10^{6}	7.0×10^{7}	nd	27.6	16.8	6.5
30	KTM 203	Clay	nd	9.0×10^{7}	nd	27.6	17.5	6.5
31	KTM 204	Clay	7.3×10^{5}	6.7×10^{8}	nd	6.7	16.8	6.7
32	KTM 208	Clay	nd	1.6×10^{10}	1.8	3.1	19.8	6.1
33	KTM 209	Clay	nd	8.1×10^{8}	5.1	13.6	19.8	6.9
34	KTM 212	Clay	nd	2.3×10^{8}	1.3	0.4	19.0	7.1
35	KTM 213	Clay	nd	6.3×10^{8}	nd	2.1	17.5	7.4
36	KTM 217	Gravel	7.6×10^{6}	6.3×10^{8}	2.5	6.1	20.1	6.9
37	KTM 218	Clay	nd	1.6×10^{9}	2.4	0.7	18.0	6.4
38	KTM 219	Clay	8.9×10^{5}	1.2×10^{9}	4.7	2.3	18.0	6.5

Nakano *et al*. Table S1

No.	Sample ID	Sample type (subsurface)	Anammox bacterial 16S rRNA gene abundance (copies L^{-1})[a]	Total bacterial 16S rRNA gene abundance (copies L^{-1})[a]	NH_4^+-N (mg L^{-1})[b]	NO_3^--N (mg L^{-1})[b]	Water Temperature (°C)[b*]	pH[b*]
39	KTM 222	Clay	nd	7.8×10^{9}	2.3	0.4	18.0	7.1
40	KTM 223	Clay	1.7×10^{6}	1.8×10^{9}	0.6	1.4	18.3	7.1
41	KTM 224	Clay	1.5×10^{6}	5.2×10^{8}	3.9	1.0	17.3	7.0
42	KTM 228	Gravel	1.6×10^{6}	1.1×10^{10}	0.6	0.6	18.4	6.5
43	KTM 229	Clay	1.7×10^{6}	1.3×10^{8}	0.2	0.2	18.8	6.6
44	KTM 230	Clay	nd	2.1×10^{8}	0.2	1.6	19.4	6.9
45	KTM 231	Clay	nd	1.9×10^{7}	0.4	0.4	18.3	6.6
46	KTM 232	Clay	nd	2.4×10^{7}	0.1	4.1	19.2	6.6
47	KTM 233	Clay	1.8×10^{6}	8.4×10^{9}	0.4	0.3	18.1	6.3
48	KTM 234	Clay	1.5×10^{6}	7.3×10^{10}	5.6	0.2	18.1	6.4
49	KTM 236	Gravel	2.8×10^{6}	1.7×10^{10}	5.0	0.6	15.5	6.9
50	KTM 241	Gravel	1.8×10^{6}	3.5×10^{8}	3.8	1.7	19.4	6.7
51	KTM 245	Clay	nd	2.7×10^{8}	0.5	0.3	19.6	6.3
52	KTM 247	Gravel	nd	1.2×10^{9}	2.2	nd	18.1	6.0
53	KTM 248	Clay	3.6×10^{5}	1.8×10^{10}	3.0	0.7	20.8	6.4
54	KTM 249	Clay	3.7×10^{6}	1.1×10^{8}	5.4	9.6	18.3	6.7
55	KTM 250	Gravel	nd	1.7×10^{8}	11.4	nd	17.5	6.6
56	KTM 251	Gravel	9.6×10^{5}	1.5×10^{8}	nd	nd	17.4	6.6
57	KTM 252	Gravel	3.3×10^{5}	5.4×10^{7}	nd	4.1	16.4	6.5
58	KTM 253	Clay	4.0×10^{5}	4.3×10^{7}	1.4	2.7	19.9	6.4

Nakano *et al*. Table S1

No.	Sample ID	Sample type (subsurface)	Anammox bacterial 16S rRNA gene abundance (copies L^{-1})[a]	Total bacterial 16S rRNA gene abundance (copies L^{-1})[a]	NH_4^+-N (mg L^{-1})[b]	NO_3^--N (mg L^{-1})[b]	Water Temperature (°C)[b*]	pH[b*]
59	KTM 258	Clay	nd	3.6×10^8	nd	24.6	18.5	6.4
60	KTM 260	Clay	nd	1.8×10^8	2.0	0.5	16.5	6.7
61	KTM 262	Clay	8.4×10^5	1.2×10^7	nd	1.7	17.6	7.0
62	KTM 263	Clay	3.0×10^5	2.1×10^8	1.6	0.6	18.9	6.6
63	KTM 264	Gravel	1.9×10^6	3.9×10^8	nd	3.9	18.4	6.6
64	KTM 265	Clay	nd	2.9×10^8	2.7	4.3	16.7	6.3
65	KTM 267	Clay	nd	1.0×10^8	0.3	1.1	16.9	6.9
66	KTM 268	Gravel	3.8×10^5	4.0×10^8	nd	0.3	17.6	7.3
67	KTM 269	Clay	5.4×10^5	2.4×10^9	0.5	0.7	20.1	6.8
68	KTM 270	Clay	8.8×10^6	9.2×10^8	0.3	0.1	20.2	6.5

[a] Data obtained in this study.

[b] Data cited from a previous report (Shakya *et al*., 2019).

[b*] Data obtained in a study by Shakya *et al*., 2019.

Clay = Clay subsurface

Gravel = Gravel subsurface

nd = not detected.

Table S2. Physicochemical properties of groundwater samples collected from shallow dug wells in the wet season

No.	Sample ID	Sample type (subsurface)	Anammox bacterial 16S rRNA gene abundance (copies L^{-1})[a]	Total bacterial 16S rRNA gene abundance (copies L^{-1})[a]	NH_4^+-N (mg L^{-1})[b]	NO_3^--N (mg L^{-1})[b]	Water temperature (°C)[b*]	pH[b*]
1	KTM 288	Gravel	nd	5.3×10^8	nd	0.4	23.9	6.2
2	KTM 290	Gravel	1.1×10^6	5.3×10^8	0.7	4.7	24.5	6.7
3	KTM 291	Gravel	1.2×10^6	2.8×10^8	nd	5.1	25.0	6.7
4	KTM 292	Gravel	nd	2.6×10^9	0.1	9.1	24.6	6.4
5	KTM 294	Gravel	nd	4.3×10^7	2.1	4.8	24.0	6.8
6	KTM 295	Gravel	2.6×10^5	5.8×10^8	2.5	12.3	23.7	6.9
7	KTM 296	Clay	nd	3.3×10^6	0.8	6.8	21.3	6.6
8	KTM 304	Gravel	4.0×10^5	5.5×10^9	0.3	0.2	23.8	7.3
9	KTM 305	Gravel	2.8×10^5	7.6×10^6	2.0	0.2	24.0	6.4
10	KTM 306	Clay	nd	7.9×10^9	nd	31.7	23.1	6.6
11	KTM 307	Clay	7.1×10^5	3.6×10^8	3.4	53.1	25.5	6.4
12	KTM 308	Gravel	7.1×10^5	4.4×10^8	nd	0.6	25.5	6.8
13	KTM 309	Gravel	3.2×10^5	5.0×10^7	nd	0.4	24.4	6.5
14	KTM 310	Gravel	nd	6.7×10^6	nd	0.5	27.9	6.3
15	KTM 311	Gravel	nd	4.1×10^7	0.1	12.3	25.6	6.5
16	KTM 318	Clay	5.4×10^5	5.3×10^8	0.4	41.0	23.1	6.0
17	KTM 320	Clay	1.4×10^5	1.6×10^8	nd	32.4	25.7	6.1
18	KTM 321	Clay	nd	2.7×10^8	0.3	10.2	26.9	6.1

Nakano *et al*. Table S2

No.	Sample ID	Sample type (subsurface)	Anammox bacterial 16S rRNA gene abundance (copies L^{-1})[a]	Total bacterial 16S rRNA gene abundance (copies L^{-1})[a]	NH_4^+-N (mg L^{-1})[b]	NO_3^--N (mg L^{-1})[b]	Water temperature (°C)[b*]	pH[b*]
19	KTM 322	Clay	1.9×10^5	5.0×10^8	2.1	21.1	23.1	6.6
20	KTM 323	Clay	nd	1.1×10^9	1.4	9.7	23.7	6.8
21	KTM 324	Clay	2.9×10^6	6.6×10^7	2.4	0.6	26.3	7.0
22	KTM 326	Clay	3.1×10^6	2.5×10^8	0.7	1.7	25.6	6.7
23	KTM 328	Clay	3.6×10^5	1.9×10^9	0.6	0.2	22.7	6.2
24	KTM 330	Clay	2.4×10^5	1.5×10^9	4.9	15.0	23.3	6.8
25	KTM 332	Gravel	2.2×10^5	2.1×10^7	nd	2.3	24.9	6.2
26	KTM 333	Gravel	3.9×10^5	5.6×10^9	1.2	0.6	26.6	6.7
27	KTM 334	Gravel	3.3×10^5	5.0×10^8	1.0	21.6	24.9	6.0
28	KTM 336	Gravel	1.7×10^5	1.6×10^9	0.1	3.9	26.3	6.4
29	KTM 337	Clay	5.9×10^5	2.0×10^7	2.4	1.5	26.1	6.6
30	KTM 339	Clay	nd	3.5×10^9	0.2	2.8	21.7	6.2
31	KTM 340	Gravel	5.2×10^5	1.3×10^9	0.6	4.4	22.4	5.8
32	KTM 342	Gravel	nd	3.1×10^8	nd	3.9	25.1	6.0
33	KTM 348	Gravel	3.3×10^5	3.6×10^6	0.4	6.1	21.5	6.7
34	KTM 351	Clay	4.2×10^5	5.0×10^8	nd	16.0	22.2	6.4
35	KTM 352	Clay	3.4×10^5	5.7×10^8	0.6	4.5	23.6	7.2
36	KTM 353	Gravel	7.2×10^5	1.0×10^{11}	3.6	8.8	21.1	7.1
37	KTM 355	Clay	1.0×10^6	2.3×10^9	4.0	0.3	21.2	7.1
38	KTM 360	Clay	2.9×10^5	2.5×10^7	nd	0.7	26.3	6.3

Nakano *et al*. Table S2

No.	Sample ID	Sample type (subsurface)	Anammox bacterial 16S rRNA gene abundance (copies L^{-1})[a]	Total bacterial 16S rRNA gene abundance (copies L^{-1})[a]	NH_4^+-N (mg L^{-1})[b]	NO_3^--N (mg L^{-1})[b]	Water temperature (°C)[b*]	pH[b*]
39	KTM 362	Clay	nd	4.2×10^9	0.1	7.1	27.2	6.6
40	KTM 363	Clay	nd	2.1×10^9	0.4	3.0	29.6	6.5
41	KTM 364	Clay	2.0×10^5	1.5×10^9	nd	32.7	32.0	6.4
42	KTM 365	Clay	nd	5.2×10^9	nd	28.2	29.4	6.5
43	KTM 366	Clay	nd	2.8×10^8	nd	23.2	22.6	6.7
44	KTM 367	Clay	nd	4.6×10^9	nd	45.5	25.2	6.5
45	KTM 368	Clay	1.3×10^5	1.7×10^9	0.5	0.5	30.4	6.7
46	KTM 369	Clay	nd	9.4×10^9	1.3	4.0	31.7	6.4
47	KTM 370	Clay	4.7×10^5	1.2×10^9	3.6	0.2	31.4	6.6
48	KTM 377	Gravel	2.3×10^5	6.4×10^9	0.2	6.3	23.8	6.5
49	KTM 378	Gravel	2.0×10^5	4.5×10^9	1.8	4.1	23.4	6.6
50	KTM 379	Clay	1.6×10^5	7.7×10^9	nd	1.9	24.5	7.1
51	KTM 381	Gravel	1.6×10^5	7.7×10^9	0.7	19.6	25.1	7.0
52	KTM 382	Clay	4.9×10^5	6.3×10^8	1.3	24.0	24.5	6.9
53	KTM 383	Clay	1.9×10^5	1.3×10^8	0.6	60.3	22.6	6.4
54	KTM 384	Clay	1.9×10^5	1.7×10^9	1.8	0.8	21.2	6.7
55	KTM 388	Gravel	nd	7.4×10^8	0.2	8.5	22.5	7.1
56	KTM 389	Clay	nd	7.1×10^9	nd	3.5	24.5	7.3
57	KTM 390	Gravel	2.3×10^5	1.9×10^8	0.9	17.6	21.2	6.7
58	KTM 394	Clay	nd	1.1×10^8	nd	39.8	23.8	6.5

Nakano *et al*. Table S2

No.	Sample ID	Sample type (subsurface)	Anammox bacterial 16S rRNA gene abundance (copies L^{-1})[a]	Total bacterial 16S rRNA gene abundance (copies L^{-1})[a]	NH_4^+-N (mg L^{-1})[b]	NO_3^--N (mg L^{-1})[b]	Water temperature (°C)[b*]	pH[b*]
59	KTM 395	Clay	nd	1.3×10^{7}	nd	22.5	21.6	6.7
60	KTM 397	Clay	1.5×10^{5}	4.8×10^{9}	nd	4.4	22.5	7.2
61	KTM 398	Clay	nd	3.0×10^{10}	1.4	nd	25.1	7.2
62	KTM 399	Clay	nd	7.2×10^{9}	nd	7.4	24.2	6.8
63	KTM 400	Clay	nd	1.6×0^{10}	nd	0.8	23.3	7.2
64	KTM 402	Clay	2.3×10^{5}	2.3×10^{9}	nd	39.0	23.8	7.1
65	KTM 403	Clay	1.4×10^{5}	2.4×10^{9}	2.4	14.9	24.4	7.1
66	KTM 405	Clay	nd	4.4×10^{9}	nd	10.5	23.3	7.5
67	KTM 406	Gravel	nd	1.5×10^{8}	0.4	nd	23.9	7.1
68	KTM 409	Clay	nd	2.6×10^{9}	0.7	3.9	26.1	6.2
69	KTM 410	Clay	1.1×10^{5}	3.3×10^{9}	nd	0.8	26.5	6.2
70	KTM 412	Clay	nd	5.7×10^{9}	nd	8.9	23.3	6.6
71	KTM 413	Clay	1.5×10^{5}	9.8×10^{8}	nd	0.3	22.1	5.7
72	KTM 415	Clay	nd	8.3×10^{9}	nd	2.2	25.7	6.9
73	KTM 418	Clay	nd	1.4×10^{9}	nd	10.3	22.4	6.7
74	KTM 419	Clay	nd	7.9×10^{9}	0.2	13.7	22.8	7.1
75	KTM 420	Gravel	nd	2.1×10^{8}	0	9.8	19.9	6.3
76	KTM 422	Clay	2.3×10^{5}	4.0×10^{9}	3.5	48.4	21.7	6.8
77	KTM 423	Clay	4.5×10^{4}	7.9×10^{9}	0.1	1.2	22.8	6.8
78	KTM 424	Clay	nd	7.9×10^{9}	0.2	3.6	23.3	7.2

Nakano *et al*. Table S2

No.	Sample ID	Sample type (subsurface)	Anammox bacterial 16S rRNA gene abundance (copies L^{-1})[a]	Total bacterial 16S rRNA gene abundance (copies L^{-1})[a]	NH_4^+-N (mg L^{-1})[b]	NO_3^--N (mg L^{-1})[b]	Water temperature (°C)[b*]	pH[b*]
79	KTM 425	Clay	1.9×10^5	1.1×10^{10}	1.0	4.7	24	7.3
80	KTM 426	Clay	nd	7.9×10^9	1.0	1.7	22.3	6.9
81	KTM 431	Clay	4.3×10^4	5.0×10^7	0	24.9	22.7	6.6
82	KTM 432	Gravel	1.2×10^5	1.4×10^9	0	13.0	24.5	6.7
83	KTM 448	Clay	5.7×10^5	2.5×10^9	3.0	1.8	23.1	6.5
84	KTM 467	Gravel	4.7×10^5	1.1×10^9	0.2	8.4	23.4	7.0

[a] Data obtained in this study.

[b] Data cited from the previous report (Shakya *et al*., 2019).

[b*] Data obtained in a study by Shakya *et al* (2019).

Clay = Clay subsurface

Gravel = Gravel subsurface

nd = not detected.

Table S3. Summary of the PCR condition used in this study

Primer	PCR condition			Cycles
	Denaturation	Annealing	Extension	
AMX-368F<br>Amx-667R	95°C, 15 s	57°C, 30 s	72°C, 20 s	35
Eub-515F<br>Eub-806R	94°C, 15 s	55°C, 30 s	72°C, 20 s	35
Pla46F<br>Amx820R	98°C, 5 s	55°C, 5 s	72°C, 10 s	35

Table S4. Physicochemical properties of shallow groundwater samples from Khumaltar and Nayachok

Season	Sample type	Abundance of anammox bacterial 16S rRNA gene (copies L^{-1})[a]	Abundance of total bacterial 16S rRNA gene (copies L^{-1})[a]	NH_4^+-N concn (mg L^{-1})[b]	NO_3^--N concn (mg L^{-1})[b]	Water temp (°C)[b*]	pH[b*]
Dry season	Nayachok (Gravel subsurface)	6.0×10^5	1.8×10^9	1.8	13.4	17.8	6.4
(February to March 2016)	Khumaltar (Clay subsurface)	2.3×10^6	1.4×10^8	nd	25.6	19.0	6.8
Wet season	Nayachok (Gravel subsurface)	1.1×10^6	5.3×10^8	0.7	4.7	24.5	6.7
(August to September 2016)	Khumaltar (Clay subsurface)	4.9×10^5	6.3×10^8	1.3	24.0	24.5	6.9

[a] Data obtained in this study.

[b] Data cited from a previous report (Shakya *et al*., 2019).

[b*] Data obtained in a study by Shakya *et al*., 2019.

nd =not detected.

Table S5. Correlation analysis results for anammox bacterial gene abundance and groundwater properties in each area and season

Season	Sample type	Correlation coefficient*				
		Abundance of total bacterial 16S rRNA gene	NH_4^+-N concn	NO_3^--N concn	Water temp	pH
Dry season	Gravel subsurface	0.448	–0.220	0.284	0.304	0.037
	Clay subsurface	0.087	0.167	0.109	–0.050	–0.251
Wet season	Gravel subsurface	0.265	0.242	0.090	–0.040	0.161
	Clay subsurface	–0.259	0.430	0.090	–0.001	–0.109
Total		0.045	0.129	0.065	0.010	–0.107

*Correlation coefficient was analyzed by R statistical software 3.6.0 (R Development Core Team, 2019) using all samples.

Table S6. RFLP groups detected in this study and their related authentic species based on 16S rRNA gene sequence

RFLP group	No. of clones				Most closely related bacterial 16S rRNA gene sequence (Accession No.)	Identity (%)	Phylum	Length (bp)	Cluster group
	Dry season		Wet season						
	Nayachok	Khumaltar	Nayachok	Khumaltar					
1	1	8		5	"*Candidatus* Brocadia fulgida" clone Y1_Summer_8 (KU217539)	98.9	*Planctomycetes*	755	Cluster 1
2		1			"*Candidatus* Brocadia fulgida" clone Y4_Summer_38 (KU217679.1)	90.2	*Planctomycetes*	736	Cluster 5
3	1	1			"*Candidatus* Brocadia fulgida" clone Y4_Summer_38 (KU217679.1)	90.2	*Planctomycetes*	737	Cluster 5
4		1			"*Candidatus* Brocadia fulgida" clone Y1_Summer_16 (KU217560.1)	98.4	*Planctomycetes*	740	Cluster 1
5		1			"*Candidatus* Brocadia caroliniensis" clone W-DXG-33 (JQ889546.1)	89.9	*Planctomycetes*	710	Cluster 5
6		1			"*Candidatus* Brocadia fulgida" clone Y1_Summer_8 (KU217539.1)	98.7	*Planctomycetes*	767	Cluster 1

Nakano *et al*. Table S6

RFLP group	No. of clones				Most closely related bacterial 16S rRNA gene sequence (Accession No.)	Identity (%)	Phylum	Length (bp)	Cluster group
	Dry season		Wet season						
	Nayachok	Khumaltar	Nayachok	Khumaltar					
7		1			"*Candidatus* Brocadia fulgida" clone Y4_Summer_16 (KU217489.1)	99.1	*Planctomycetes*	740	Cluster 1
8	1	1			"*Candidatus* Brocadia fulgida" clone Y1_Summer_16 (KU217560.1)	98.7	*Planctomycetes*	732	Cluster 1
9		3			"*Candidatus* Brocadia fulgida" clone Y1_Summer_8 (KU217539.1)	99.3	*Planctomycetes*	692	Cluster 1
10		1			"*Candidatus* Brocadia fulgida" clone Y4_Summer_38 (KU217679.1)	90.2	*Planctomycetes*	737	Cluster 5
11		1			"*Candidatus* Brocadia fulgida" clone Y1_Summer_8 (KU217539.1)	90.1	*Planctomycetes*	721	Cluster 1
12		2			"*Candidatus* Brocadia fulgida" clone Y1_Summer_8 (KU217539.1)	99.6	*Planctomycetes*	742	Cluster 1
13		1			"*Candidatus* Brocadia fulgida" clone S-DXG-25 (JQ889388.1)	89.1	*Planctomycetes*	680	Cluster 6

Nakano *et al*. Table S6

RFLP group	No. of clones				Most closely related bacterial 16S rRNA gene sequence (Accession No.)	Identity (%)	Phylum	Length (bp)	Cluster group
	Dry season		Wet season						
	Nayachok	Khumaltar	Nayachok	Khumaltar					
14		1			"*Candidatus* Brocadia fulgida" clone Y1_Summer_16 (KU217560.1)	98.6	*Planctomycetes*	732	Cluster 1
15		1			"*Candidatus* Brocadia fulgida" clone Y1_Summer_8 (KU217539.1)	98.7	*Planctomycetes*	740	Cluster 1
16		1			"*Candidatus* Scalindua marina" clone S-FZ-39 (KM925797.1)	89.0	*Planctomycetes*	689	Cluster 5
17		1			"*Candidatus* Brocadia fulgida" clone Y1_Summer_8 (KU217539.1)	99.6	*Planctomycetes*	740	Cluster 1
18		1	4		"*Candidatus* Anammoxoglobus propionicus" clone Y2_Summer_18 (KU217830.1)	97.8	*Planctomycetes*	741	Anammoxoglobus
19		1			"*Candidatus* Brocadia fulgida" clone Y1_Summer_16 (KU217560.1)	98.9	*Planctomycetes*	754	Cluster 1

Nakano *et al*. Table S6

RFLP group	No. of clones				Most closely related bacterial 16S rRNA gene sequence (Accession No.)	Identity (%)	Phylum	Length (bp)	Cluster group
	Dry season		Wet season						
	Nayachok	Khumaltar	Nayachok	Khumaltar					
20		1			"*Candidatus* Brocadia fulgida" clone Y4_Summer_38 (KU217679.1)	90.1	*Planctomycetes*	751	Cluster 5
21		1			"*Candidatus* Brocadia fulgida" clone Y1_Summer_16 (KU217560.1)	98.5	*Planctomycetes*	764	Cluster 1
22		1			"*Candidatus* Anammoxoglobus propionicus" clone Y2_Summer_21 (KU217773.1)	90.1	*Planctomycetes*	744	Cluster 6
23	32		7	7	"*Candidatus* Scalindua marina" clone S-FZ-39 (KM925797.1)	97.9	*Planctomycetes*	768	Cluster 2
24	1				"*Candidatus* Scalindua marina" clone S-FZ-39 (KM925797.1)	99.3	*Planctomycetes*	747	Cluster 3
25	5		5		"*Candidatus* Anammoxoglobus propionicus" clone Y2_Winter_13 (KU217791.1)	97.4	*Planctomycetes*	744	Cluster 4

Nakano *et al*. Table S6

RFLP group	No. of clones				Most closely related bacterial 16S rRNA gene sequence (Accession No.)	Identity (%)	Phylum	Length (bp)	Cluster group
	Dry season		Wet season						
	Nayachok	Khumaltar	Nayachok	Khumaltar					
26			12	14	"*Candidatus* Brocadia fulgida" clone Y2_Summer_35 (KU217482.1)	99.5	*Planctomycetes*	781	Cluster 1
27			1		"*Candidatus* Anammoxoglobus propionicus" clone Y2_Winter_13 (KU217791.1)	96.3	*Planctomycetes*	767	Cluster 4
28			2		"*Candidatus* Anammoxoglobus propionicus" clone Y2_Winter_13 (KU217791.1)	98.3	*Planctomycetes*	766	Cluster 4
29			2		"*Candidatus* Brocadia fulgida" clone Y4_Winter_57 (KU217750.1)	99.3	*Planctomycetes*	771	Brocadia
30			4	2	"*Candidatus* Brocadia fulgida" clone Y1_Winter_37 (KU217511.1)	99.5	*Planctomycetes*	766	Cluster 1
31			2	2	"*Candidatus* Brocadia fulgida" clone Y2_Summer_35 (KU217482.1)	99.8	*Planctomycetes*	785	Cluster 1

Nakano *et al*. Table S6

RFLP group	No. of clones				Most closely related bacterial 16S rRNA gene sequence (Accession No.)	Identity (%)	Phylum	Length (bp)	Cluster group
	Dry season		Wet season						
	Nayachok	Khumaltar	Nayachok	Khumaltar					
32				1	"*Candidatus* Brocadia fulgida" clone AmxW-WSK-17 (JX243636.1)	98.6	*Planctomycetes*	721	Brocadia
Total	41	32	39	31					
